# Inflammatory Role of TLR-MyD88 Signaling in Multiple Sclerosis

**DOI:** 10.3389/fnmol.2019.00314

**Published:** 2020-01-10

**Authors:** Chao Zheng, Jingtao Chen, Fengna Chu, Jie Zhu, Tao Jin

**Affiliations:** ^1^Department of Neurology and Neuroscience Center, The First Hospital of Jilin University, Changchun, China; ^2^Institute of Translational Medicine, The First Hospital of Jilin University, Changchun, China; ^3^Department of Neurobiology, Care Sciences and Society, Karolinska Institute, Karolinska University Hospital Huddinge, Stockholm, Sweden

**Keywords:** multiple sclerosis, experimental autoimmune encephalomyelitis, Toll-like receptors, myeloid differentiation primary response protein 88, inflammation

## Abstract

Multiple sclerosis (MS) is a neuro-autoimmune and neurodegenerative disorder leading to chronic inflammation, demyelination, axonal, and neuronal loss in the central nervous system (CNS). Despite intense research efforts, the pathogenesis of MS still remains unclear. Toll-like receptors (TLRs) are a family of type I transmembrane receptors that play a crucial role in the innate immune response. Myeloid differentiation factor 88 (MyD88) is the adaptor of major TLRs. It has been widely considered that the TLR-MyD88 signaling pathway plays an important role in the occurrence and development of autoimmune disease. Data have revealed that the TLR-MyD88 signaling may be involved in the pathogenesis of MS and experimental autoimmune encephalomyelitis (EAE), an animal model for MS, by regulating the antigen presentation of dendritic cells, the integrity of blood-brain barrier (BBB), and the activation of T cells and B cells. Here, we summarize the role of TLRs and MyD88 in MS and discuss the possible therapies that are based on these molecules.

## Introduction

Multiple sclerosis (MS) is a neuro-autoimmune and neurodegenerative disorder leading to chronic inflammation, demyelination, axonal, and neuronal loss in the central nervous system (CNS). There are over 2 million people worldwide suffering from this disease. The generation and development of MS are heterogeneous and manifold (Kipp et al., 2017). MS can be divided into four types: relapsing-remitting MS (RRMS), primary progressive MS (PPMS), secondary progressive MS (SPMS) and progressive-relapsing MS (PRMS; Fitzpatrick and Downer, 2017). Most patients with MS have a poor prognosis after recurrent remission and relapse several times. Pathological observation shows that the lesions of patients with MS are characterized by multifocal areas of myelin sheath destruction, oligodendrocyte death, and axonal and neuronal damage. The demyelination in CNS results in a series of clinical symptoms, such as paresthesia, ataxia, cognitive impairment, and loss of vision and mobility (Lassmann and Bradl, 2017). Most patients affected by MS are young adults, and the effect of MS at the peak of their active life brings enormous physical, psychosocial, and economic burdens to their families and society.

It is generally assumed that MS is associated with both environmental and inherited factors. Besides, various types of immune cells such as dendritic cells, macrophages, monocytes, myelin-specific T lymphocytes, B cells, activated microglia, and reactive astrocytes are essential in disease pathogenesis (Danikowski et al., 2017). However, the pathogenesis of MS remains unclear. Currently, there are eight drugs approved by the American Food and Drug Administration (FDA) for the treatment of MS, but their therapeutic effects vary among individuals (Vargas and Tyor, 2017). Therefore, the pathogenesis of MS and therapeutic strategies for its treatment still merit in-depth study.

It is well documented that there are several signaling molecules, such as the toll-like receptors (TLRs), that are involved in MS pathogenesis and could become future therapeutic targets for the disease (Liu et al., 2014; Nyirenda et al., 2015). TLRs are a family of receptors involved in pathogen recognition and host defense. They localize on the surface or in the endosomes of several immune-relevant cell types, such as macrophages, dendritic cells, T cells, B cells, astrocytes, oligodendrocytes, epithelial cells, and endothelial cells (Farrugia and Baron, 2017). Myeloid differentiation primary response protein 88 (MyD88) is an adaptor that connects TLRs to downstream molecules (Farrugia and Baron, 2017). It has been reported that the TLR-MyD88 signaling pathway contributes to the pathogenesis of neurological diseases such as MS (Reynolds et al., 2012; Robinet et al., 2017). Here, we review and summarize the currently available literature about the role of TLR and MyD88 in MS and highlight the therapies targeting TLR and MyD88 that may offer future therapeutic possibilities.

## Structures and Biological Functions of TLRs and MyD88

**Figure 1:**
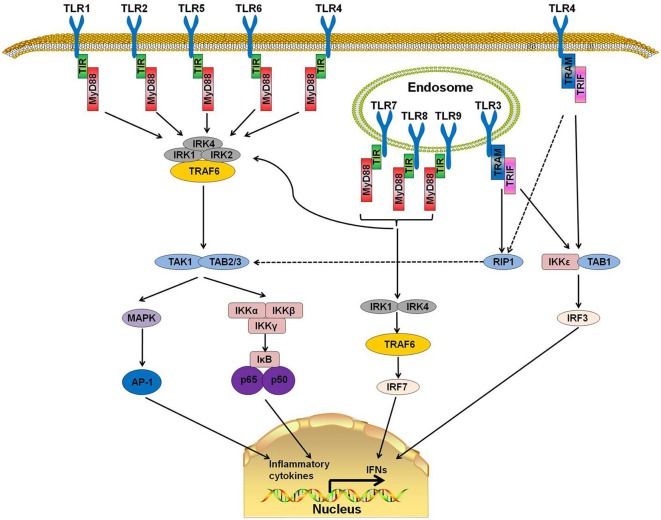
Toll-like receptor (TLR) signaling pathways. TLR 1, 2, 4, 5, and 6 are located on the cell surfaces. When stimulated by pathogen-associated molecular patterns (PAMPs), myeloid differentiation primary response protein 88 (MyD88) interacts with IL-1 receptor-associated kinase-4 (IRAK-4) and forms a MyD88-IRAK-4 complex, which recruits IRAK-1 and IRAK-2, resulting in the phosphorylation of IRAKs. IRAKs leave MyD88 after phosphorylation and interact with tumor necrosis factor receptor-associated factor 6 (TRAF6). TRAF6 induces the activation of TAK-1 and TAB2/3 following consequent activation of I-κB (IκB) and mitogen-activated protein kinase (MAPK). The activation of IκB and MAPK result in the subsequent translocation of nuclear factor-κB (NF-κB) and AP-1 to the nucleus. TLR3, 7, 8, and 9 are on the endosome. Stimulation of these TLRs leads to the recruitment of MyD88, IRAK4, IRAK1, and TRAF6 and the translocation of interferon-regulatory factor 7 (IRF7). This signaling cascade leads to the production of interferons (IFNs). TLR3 and part of TLR4 use TIR-domain-containing adapter-inducing interferon-β (TRIF)as their adaptor. The interaction of TRIF with receptor interacting protein 1 (RIP1) leads to RIP1 polyubiquitination and their combination with TAB2 and TAB3, which can result in the translocation of NF-κB and AP-1 to the nucleus. Moreover, TRIF can also induce the production of IFNs by activating the I-κB Kinase/TANK-binding kinase 1 (IKK$\rvarepsilon$/TBK1) complex and IRF3.

TLRs are a family of type I transmembrane receptors that play a crucial role in the innate immune response (Li J. et al., 2013). They can recognize a wide variety of bacterial, fungal, protozoan, and viral components and can activate the immune response. They are abundantly expressed on several cell types related to the immune response, such as macrophages, dendritic cells, T cells, B cells, and epithelial cells (Fernández-Paredes et al., 2017). Currently, 10 TLRs have been found in humans (TLR1-10) and 12 in mice (TLR1-9, TLR11–13; Ntoufa et al., 2016). TLRs are divided into two groups according to their location. TLR 1, 2, 4, 5, 6, and 10 are localized on the cell surface, and TLRs 3, 7, 8, and 9 are localized within intracellular compartments (Figure 1).

TLRs have a structure similar to other type 1 transmembrane glycoprotein receptors. The extracellular domain is a leucine-rich repeat N-terminal domain that has the ability to detect a wide range of pathogen-associated molecular patterns (PAMPs) and endogenous danger-associated molecular patterns (DAMPs; Ntoufa et al., 2016). The cytoplasmic Toll/Interleukin-1 receptor (TIR) domain, which is always present at the C-terminal domain, is a conserved interleukin-1 receptor (IL-1R) family that binds with downstream adaptor molecules (Gambuzza et al., 2011).

MyD88, a downstream adaptor protein of TLR, is critical in the signal transduction of the TLR signaling pathway. The TLR signaling is mainly divided into MyD88-dependent and MyD88-independent pathways. With the exception of TLR3, all TLRs mediate the downstream signaling pathway *via* MyD88 (Falck-Hansen et al., 2013). MyD88 protein contains two domains: a Toll/IL-1R Interleukin-1 receptor (TIR) domain and a death domain (DD). TLRs have been identified as ligands of several kinds of microbial and endogenous molecules (Jiménez-Dalmaroni et al., 2016). When TLRs recognize PAMPs or DAMPs, the DD of MyD88 interacts with the DD of IL-1 receptor-associated kinase-4 (IRAK-4) and forms the MyD88-IRAK-4 complex, which recruits IRAK-1 and IRAK-2, resulting in the phosphorylation of IRAKs. IRAKs leave MyD88 after phosphorylation and interact with tumor necrosis factor receptor-associated factor 6 (TRAF6; Xiang et al., 2015). TRAF6 then induces the activation of TGF-β activated kinase-1 (TAK-1) and TAK1-binding proteins (TAB) 2 and 3, which consequently activate the nuclear factor-κB (NF-κB) signaling pathway by phosphorylating I-κB (IκB). Phosphorylation of IκB results in the ubiquitylation and degradation of itself and the subsequent release and translocation of NF-κB to the nucleus (Kawai and Akira, 2007, 2010; Kumar et al., 2011). Additionally, TAK-1 can also activate c-Jun N-terminal kinase (JNK), mitogen-activated protein kinase (MAPK) and Phosphatidylinositol 3-Kinases (PI3K). The activation of these downstream kinases and pathways leads to a cascade of inflammatory responses (Xiang et al., 2015).

MyD88 is the canonical downstream adaptor of most TLRs (Deguine and Barton, 2014). *MyD88*-deficient mice have been used widely to explore the role of TLRs (Takeuchi et al., 2000; Scanga et al., 2002). *MyD88*-deficient mice were reported to lack a response to lipopolysaccharide (LPS; a ligand of TLR4) and were insusceptible to endotoxic shock (Kawai et al., 1999). In human, several MyD88-inactivating mutations have been found in patients with recurrent infections (von Bernuth et al., 2008) or different kinds of malignancies (Wang et al., 2014). MyD88 promotes the production of type I interferon by phosphorylating the transcription factor interferon-regulatory factor 7 (IRF7; Honda et al., 2004; Ning et al., 2011). These reports indicate that TLR-MyD88 signaling is an important trigger in inflammatory responses and that its abnormal functioning may cause autoimmune diseases or immunodeficiency.

## MS and Experimental Autoimmune Encephalomyelitis

MS is a chronic inflammatory disease that mainly occurs in young adults. The damage of myelin, axons, and neurocytes in the CNS is the most typical histopathological feature of patients with MS (Hewer et al., 2013; Grigoriadis et al., 2015). In most patients, the disease starts at the peak of their life, with phases of remissions and relapses. The patients present with cognitive impairment, extreme fatigue, and paralysis after relapsing several times (Legroux and Arbour, 2015). MS is a disease that has a negative impact on young adults and imposes burdens on their families and society. Even though MS has been studied extensively, its pathogenesis remains unclear, and further studies are required to elucidate MS pathogenesis and to develop effective treatment strategies (Xie et al., 2017).

Based on the inflammatory response in MS, experimental autoimmune encephalomyelitis (EAE) animal models have been generated to reproduce this disease in animals. Even though there are many kinds of animal models of MS, such as the Theiler’s murine encephalomyelitis virus model and the cuprizone model, EAE is the most accepted and widely used animal model for studying MS (Lassmann and Bradl, 2017). Models of EAE can be induced in a variety of vertebrates. Mice, rats, and primates are the most common animals used to study the pathogenesis and therapy of MS. To induce EAE in C57BL/6 mice, myelin oligodendrocyte glycoprotein (MOG) accompanied with mycobacterium-containing complete Freud’s adjuvant (CFA) and pertussis toxin (PTX) are administrated to the animal (Kipp et al., 2017). These reagents enhance the autoimmune responses against CNS myelin through the action of immune cells, cytokines, and signaling molecules (Lucchinetti et al., 2000; Tran et al., 2000). Both myelin damage and axonal loss are observed in EAE animal models (Kipp et al., 2017).

## Association Between the Pathogenic Factors and the TLR-MyD88 Signaling Pathway in MS and EAE

**Figure 2:**
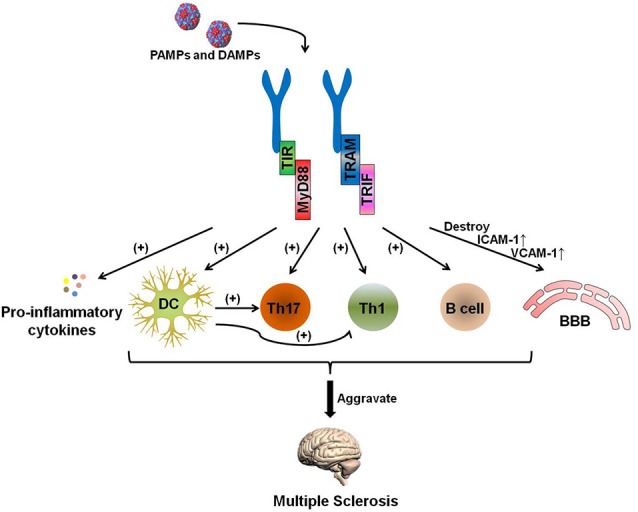
The TLR-MyD88 signaling pathway contributes to the pathogenesis of multiple sclerosis (MS). The TLR-MyD88 and TLR-TRIF signaling pathways activate dendritic cells (DCs), Th17 cells, Th1 cells, and B cells and increase pro-inflammatory cytokine secretion. These pathogenic DCs can also promote the activation of Th1 and Th17 cells. Furthermore, the TLR signaling pathway destroys the blood brain barrier (BBB) and increases the expression of BBB-expressed adhesion molecules [ICAM-1 and vascular cell adhesion molecules (VCAM)-1] after being stimulated by PAMPs and endogenous danger-associated molecular patterns (DAMPs). The activation of these cells and the destruction of the BBB aggravate MS.

The pathogenesis of MS is complicated, and certain uncertainties remain to be explored. Experimental data have shown that the development of MS can generally be attributed to the following process: auto-antigen-specific T cells are activated by antigen-presenting cells (APCs) in the periphery. These pathogenic lymphocytes migrate through the blood-brain barrier (BBB) with the help of adhesion molecules and infiltrate into the lesion to attack the myelin and, subsequently, the axon (Grigoriadis et al., 2015; Figure 2).

### Pathogenic T Cells

The polarization of naive CD4^+^ T cells into T helper cells (Th cells) is an important process in the development of MS and is regulated by diverse factors. TLR is one of the necessary factors for the activation of autoreactive T cells (Hansen et al., 2006). Several studies in both human and animal models have shown that Th17 cells have a pathogenic role in MS and EAE (Tzartos et al., 2008; Brucklacher-Waldert et al., 2009). The frequency of Th17 cells is significantly higher in the cerebrospinal fluid (CSF) of patients with MS than in healthy controls. In addition, Th17 cells secrete several kinds of pro-inflammatory cytokines, such as IL-17, IL-21, IL-22, IL-23, and TNF-α, that promote inflammatory responses (Becher and Segal, 2011). IL-17 is a cytokine produced mainly by Th17 cells. IL-17 is able to induce the secretion of IL-1β, IL-6, and nitric oxide and mediates BBB dysfunction during MS (Jadidi-Niaragh and Mirshafiey, 2011). High levels of IL-17 have been observed in demyelinating plaques of the CNS, blood, and CSF of patients with MS (Kostic et al., 2015).

Th1 cells, which can be found in lesions in the CNS in EAE, are also one of the main pathogenic T cells in MS and EAE. Adoptive transfer of myelin-specific Th1 cells into mice induced severe EAE, and IFN-γ, a pro-inflammatory cytokine that can be produced by Th1 cells, was detected at a high level in the CSF of patients with MS (Mastorodemos et al., 2015).

The differentiation and functions of Th17 can be regulated by the TLR-MyD88 signaling pathway. The TLR ligand can induce Th17 and Th1 differentiations *via* the IL-6/TGF-β-mediated pathway and IL-12 or IFN-α, respectively (Shi et al., 2013). IL-17 levels increased in MOG_37–50_-specific CD4^+^ T cells and unprimed CD8^+^ T cells when stimulated by TLR agonists, such as LPS, CpG, and curdlan (Steckner et al., 2016). CD4^+^CD25hi FOXP3^+^ regulatory T cells (Tregs) are a cell type that maintains immune tolerance during MS. Nyirenda et al. (2015) stimulated Tregs from MS patients with Pam3Cys (an agonist of TLR1/2) and found that Pam3Cys reduced their suppressive function and skewed them into a Th17-like phenotype. Additionally, activation of TLR-MyD88 results in a signaling transduction cascade, which finally promotes the translocation of NF-κB into the nucleus (Kawai and Akira, 2007). NF-κB mediates the secretion of IL-6, which can promote the differentiation of Th17 (Jadidi-Niaragh and Mirshafiey, 2011; Karin and Wildbaum, 2015). Furthermore, the activation of NF-κB can also induce the release of reactive oxygen species and cause neuronal vulnerability. Moreover, Reynolds et al. (2010) found that deficiency of TLR2 in Th17 cells reduced their ability to trigger EAE. In addition, *MyD88*^−/−^ CD4^+^ T cells showed an impairment in IL-17A production and Th17 cell differentiation because of the reduction of mTOR activation (Chang et al., 2013). It has also been reported that MyD88 is necessary for the differentiation of splenic Th17 cells (Marta et al., 2009). Taken together, these data show that Th1 and Th17 cells are powerful contributors to the pathogenesis of MS and EAE and that the TLR-MyD88 signaling pathway plays a key role in the activation and functions of Th1 and Th17 cells.

### B Cells

An increasing body of evidence shows that B cells promote MS (Kinzel and Weber, 2016). B cells are a type of immune cell that can amplify the immune effect of T cells. B cells, being APCs, have the ability to present auto-antigens to CD4^+^ T-cells and promote Th1 and Th17 responses through their surface markers (Weissert, 2017). Some B cells undergo a proliferative phase and develop into plasma cells, which secrete myelin-specific antibodies. These antibodies are highly structure-specific and can lead to functional impairment and lesion evolvement (Nguyen et al., 2017). The intrathecal oligomeric myelin-specific antibodies have been used to diagnose MS for a long time (Nguyen et al., 2017). In addition to producing antibodies, B cells also exert their effector functions *via* cytokine secretion. Several studies have associated MS with abnormally high levels of TNF-α and lymphotoxin-α produced by B cells (Bar-Or et al., 2010). The proportion of granulocyte-macrophage colony-stimulating factor (GM-CSF)-producing B cells in patients with MS is higher than in healthy controls (Li et al., 2015).

The expression levels of TLR are different in various developmental stages of B cells, and they may relate to the functions of B cells (Marron et al., 2012). In human, Bernasconi et al. (2002) found that CpG (TLR9 agonists) could activate memory B cells. Thus, they speculated that TLR stimulation may be a mechanism for maintaining the serological memory of B cells. In mammal, mouse naive B cells could proliferate and differentiate after being stimulated by TLR agonists such as LPS and CpG, which are independent of T cells or the B cell receptor (BCR). None of the T cell subsets were able to sustain B cell proliferation in the absence of a TLR agonist (Ruprecht and Lanzavecchia, 2006). Moreover, the effector memory T cells killed naive B cells in the absence of a TLR agonist (Ruprecht and Lanzavecchia, 2006). Several studies have confirmed that the expression of TLRs is increased in brain lesions of both EAE and MS. In addition, the activation of the TLR-MyD88 signaling pathway promotes the production of pro-inflammatory cytokines, which aggravates MS. To summarize, the progression of MS is closely linked with B cells. TLR-MyD88 signaling is essential for B cell proliferation and differentiation. Therefore, TLR is an indispensable factor in the pathogenesis of MS.

### Blood-Brain Barrier

The BBB is a continuous membranous barrier that separates the CNS from the circulatory system. It is formed by specialized endothelial cells attached *via* tight junctions and adherence junctions. The BBB acts as a guard to ensure proper brain function by preventing harmful molecules from entering the CNS (Ortiz et al., 2014) and transduces signals from the vascular system to the brain. Under normal circumstances, CNS is considered as a highly regulated and active site of immune surveillance (Negi and Das, 2018). In contrast, during MS, lymphocytes from the periphery can cross the BBB and migrate into the CNS (Ellwardt and Zipp, 2014). Therefore, the disruption of the BBB is a prerequisite for the development of MS.

Activation of the TLR-MyD88 signal pathway can result in BBB destruction, and TLR inhibition can also reduce the BBB damage. Melatonin-reduced LPS induced BBB damage by inhibiting the TLR4 signaling pathway in neonatal rats (Hu et al., 2017). In TLR2^−/−^ mice, activation of the matrix metalloproteinase-9 (MMP9) in astrocytes was lower than in wild type (WT) mice, and this reduction resulted in attenuated damage to the BBB in *TLR2^−/−^* mice with intracerebral hemorrhage (Min et al., 2015).

To cross the BBB, leukocytes in the blood must adhere to the vessel wall before transendothelial migration (Alvarez et al., 2011). The process of adhesion is regulated by adhesion molecules such as intercellular adhesion molecules (ICAM), vascular cell adhesion molecules (VCAM), and leukocyte functional antigen-1 (LFA-1; Dietrich, 2002). The expression of these adhesion molecules can be regulated by the TLR-MyD88 pathway. TLR2 and TLR4 initiate the expression of ICAM-1 by activating the MyD88-dependent signaling pathway and promoting lymphocyte adhesion on the vessel wall (Cheng and Lee, 2016). The attenuated TLR signal pathway can reduce the expression of VCAM-1 and ICAM-1 on vascular endothelial cells (Bhaskar et al., 2016). In conclusion, the disruption of the BBB is a prerequisite for MS development, and the integrity of the BBB can be destroyed by the activation of the TLR signaling pathway directly or indirectly.

### Dendritic Cells

Antigen presentation is a critical event involved in the process of the activation of auto-reactive T cells. Dendritic cells (DCs), which are professional APCs, are the key controllers of innate and adaptive immunity. DCs are a population of leukocytes and are divided into plasmacytoid DCs (pDCs) and conventional DCs (cDCs; Benvenuti, 2016). Recent data suggest that DCs make substantial contributions to the pathogenesis of MS. At the onset of MS, DCs provide signals for T cell polarization. Once these auto-reactive T cells cross the BBB and enter the CNS, the resident DCs again provide the stimulating signals to reactivate these auto-reactive T cells and promote the progress of MS (Grigoriadis et al., 2015).

The functions of DCs can be altered by several factors. When cultured in the presence of specific drugs or cytokines [such as vitamin D, estrogen, andrographolide, or TGF-β (Pettersson et al., 2004; Xiao et al., 2007; Carreño et al., 2011; Xie et al., 2017)], DCs can be altered into a tolerogenic phenotype and participate in both central and peripheral tolerance (Kim and Diamond, 2015). However, when the pro-inflammatory signals are activated in DCs, DCs become aggressive, resulting in hyperfunction of the immune response. The TLR-MyD88 signaling pathway is one of these pro-inflammatory signals. Sweeney et al. (2011) reported that IL-23, IL-1β, and IL-12p40 levels were increased in the supernatants after treatment of DCs with zymosan (a stimulant of TLR-2). In pDCs, the TLR-MyD88-dependent pathway can lead to the activation of IRF-3 and IRF-7 and increase the synthesis and secretion of pro-inflammatory chemokines, such as interferon-inducible protein 10 (IP-10), RANTES, and type I interferons (INF-β and INF-α). Mycobacterium indicus pranii (MIP) is a mycobacterium that can stimulate DCs *via* TLR. MIP can up-regulate costimulatory molecules and induce the production of TNF-α, IL-12p40, and IL-6 from DCs in a MyD88-dependent manner (Kumar et al., 2015). Similarly, LPS (an agonist of TLR-4) has the same effects. When stimulated by LPS, DCs secreted high levels of IL-6 and IL-12, and the expressions of surface molecules also increased slightly (Mirzaee et al., 2015). These results indicate that the activation of the TLR-MyD88 signaling pathway can promote the evolution of DCs into a pathogenic status.

## TLR-MyD88 Signaling in MS/EAE

### TLR2

TLR2, a cell-surface TLR, is expressed by various cell types, such as DCs, T cells, B cells, mast cells, and epithelial cells (Huang and Pope, 2009). As a pattern-recognition receptor, TLR2 can bind a wide range of exogenous ligands and endogenous DAMPs (Shi et al., 2012). It has been evidenced that the TLR2 signal is involved in the pathogenesis of MS and EAE. An increased level of a TLR2 agonist was detected in patients with SPMS (Sweeney et al., 2011). Tregs expressed TLR2 at higher levels in blood from MS patients than in that of healthy controls (Nyirenda et al., 2015). EAE mice immunized with MOG_35–55_ had an increased level of TLR2 in a time-related pattern in the brain. The expression of I-κ Bα, MCP-1, and TNF-α also increased in TLR2-expressing regions in the brain (Zekki et al., 2002). Several TLR2 ligands can reduce the suppressive function of Tregs and shift human Tregs into a Th17-like phenotype. Reynolds et al. (2010) reported that TLR2 activation enhanced Th17 cell proliferation and Th17 cytokine production in mice. γδ T cells stimulated with TLR2 agonists can also increase IL-17 production (Martin et al., 2009; Reynolds et al., 2010). In addition to the activation of autoimmune T cells, the stimulation of TLR2 can also mediate the secretion of pro-inflammatory molecules. Pentraxin-3 is a protein released during inflammation that plays diverse roles in tissue injury. Increased secretion of pentraxin-3 has been reported in human microglia and macrophages with TLR2 agonist stimulation (Ummenthum et al., 2016).

Studies have shown that TLR2 deficiency protected mice against EAE (Rocca et al., 2017). TLR2 deficiency could help EAE mice tolerate extraneous antigens. The symptoms of EAE in mice worsened when WT mice with EAE were infected with respiratory pathogen *Streptococcus pneumonia*. However, the pro-inflammatory effect of *Streptococcus pneumonia* was lost in TLR2-deficient mice (Herrmann et al., 2006). The phosphorylated dihydroceramides derived from porphyromonas gingivalis strongly enhanced the clinical symptoms of WT EAE mice *via* APC and decreasing Tregs. However, they failed to play this role in TLR2-deficient mice. In the same way, the reduction of TLR2 expression directs disease toward a good prognosis. Celastrol, a pentacyclic-triterpene extracted from the roots of *Tripter-ygium wilfordii Hook*, reduced the clinical scores of EAE, possibly through the reduction of TLR2 expression in the brain (Abdin and Hasby, 2014). It has been reported that EAE mice that received TLR2^−/−^ CD4^+^ T cells by passive transferrence exhibited low clinical scores compared to mice receiving normal CD4^+^ T cells (Reynolds et al., 2010). TLR2 may have a strong capacity to induce pro-inflammatory responses and enhance EAE. However, some studies have demonstrated contradictory results. In one, TLR2 was shown not to be necessary for EAE induction, and the *TLR2*^−/−^ mice showed similar clinical symptoms as normal C57BL/6 mice with EAE (Marta et al., 2008). There is also a view that TLR2 has both pro- and anti-inflammatory effects. TLR282ile, a TLR2 variant, can promote the expansion of Treg and the polarization of T cells toward Th1/Th17 phenotypes. TLR282met, another variant of TLR2, can block the expansion of Tregs as well as reducing the secretion of IFN-γ and IL-17A (Piermattei et al., 2016).

### TLR3

TLR3 is a member of the TLR family that is expressed on the endosomes of DCs, T cells, B cells, NK cells, and macrophages (Verma and Bharti, 2017). TLR3 is the only TLR that does not activate downstream molecules through MyD88. TLR3 uses TIR-domain-containing adapter-inducing interferon-β (TRIF) as its downstream adaptor molecule (Chattopadhyay and Sen, 2014). There are also some differences between the functions of TLR3 and those of other TLRs. The activation of TLRs has therapeutic potential in autoimmune diseases (Touil et al., 2006). Touil et al. (2006) treated the EAE model with polyinosinic-polycytidylic acid (poly I:C), a double-stranded RNA agonist of TLR3, and found that poly I:C suppressed relapsing demyelination in EAE mice by inducing endogenous IFN-β and CCL2. Both peripheral blood mononuclear cells (PBMCs) from healthy controls and patients with RRMS responded only to LPS rather than poly (I:C) after stimulation with LPS and poly(I:C), respectively (Crowley et al., 2015). These results indicated that TLR3 may function completely differently than other TLRs and may play a therapeutic role in MS and EAE.

### TLR4

Similar to TLR2, TLR4 is also a surface TLR that has been demonstrated to be involved in the pathogenesis of MS. TLR4-mediated NF-κB signaling induces the transcription of pro-inflammatory genes encoding cytokines, chemokines, and enzymes (Trotta et al., 2014). TLR4 activation in CD4^+^ T cells enhances the proliferation and survival of CD4^+^ T cells, and TLR4 expression by T cells is essential for the development of EAE (Reynolds et al., 2012). TLR4 can recognize different kinds of exogenous PAMPs and endogenous ligands, such as LPS and necrotic cells. LPS acts as an adjuvant and promotes the severity of EAE. Moreover, LPS increased IL-17 secretion by stimulating fully differentiated Th17 cells *in vitro*. The phosphorylation of NF-κB in Th1 and Th17 cells was increased after stimulation with LPS. Exposure of CD4^+^CD25^+^ T cells to LPS enhanced their survival and proliferation and up-regulated their surface molecular markers, such as MHC-II, CD69, and B7–1. LPS had the ability to stimulate APCs to secrete several cytokines, such as IL-6, IL-23, INF-γ, and TGF-β. In addition, LPS, Mycobacterium tuberculosis, and PTX, which is commonly used for EAE induction, have been testified to function through the TLR4 pathway (Heldwein et al., 2003; Kerfoot et al., 2004; van de Veerdonk et al., 2010). PTX upregulates P-selectin in the CNS TLR4 signaling and results in lymphocyte accumulation. PTX is used in EAE induction to facilitate the permeabilization of the BBB and prevent autoreactive T cell anergy. PTX failed to induce lymphocyte rolling and adhesion in TLR4-deficient mice with EAE, which indicated that TLR4 should be necessary for the induction of EAE (Racke et al., 2005). In addition to PTX, another protein named Env-ms had been reported to destroy BBB and promote MS development *via* TLR4. Env-ms is an envelope protein of MS-associated retrovirus and can be found in most patients with MS. This envelope protein increases the expression of ICAM-1 adhesion molecules on brain endothelial cells. Increase in ICAM-1 can promote the adhesion of pathogenic lymphocytes to vascular endothelial cells and assist them in crossing the BBB. The pathogenic effect of Env-ms depends on TLR4, and knocking down TLR4 with small interfering RNA (siRNA) abolishes the effect of Env-ms (Duperray et al., 2015).

Knockdown of TLR4 abolished the production of inflammatory mediators by astrocytes. Even though TLR4 plays an important role in the inflammatory response and EAE induction, there are some different opinions about the role of TLR4 in MS and EAE (Marta et al., 2008). Kerfoot et al. (2004) reported that EAE was less severe in *TLR4*^−/−^ mice than in WT mice. Another study, however, showed entirely different results, finding that deficiency of TLR4 exacerbated EAE symptoms (Marta et al., 2008). Another study showed that *TLR4*^−/−^ mice exhibit an increased frequency of Th17 cells and level of serum IL-17. Moreover, the expression of IL-6 and IL-23 by TLR4^−/−^ mouse splenic mDC also increased. These results show that TLR4 has a controversial role in MS and EAE (Marta et al., 2009).

### TLR6 and TLR7

Studies on TLR6 and TLR7 in MS and EAE are limited. Both TLR2 and TLR6 form functional heterodimers. Similar to *TLR2*^−/−^ mice, *TLR6*^−/−^ mice are also susceptible to EAE. Increased expression of TLR6 and TLR7 in the spinal cord was observed in EAE mice (Prinz et al., 2006). However, IFN-β1a inhibited the secretion of Th17-polarizing cytokines in DCs from RR MS patients by up-regulating the expression of TLR7 (Zhang et al., 2009), which results in conflicting findings in animals and human. It reveals that TLR7 may have a positive effect on MS to some extent. Therefore, the effects of TLR6 and TLR7 on MS and EAE require further investigation.

### TLR8

TLR8, as a member of the TLR family, is located in intracellular endosomal compartments. The expression of TLR8 in the spinal cord was found to be increased in EAE mice (Soulika et al., 2009). 1,25-dihydroxyvitamin D3, which has a significant therapeutic effect on EAE, could reduce the expression of TLR8 in the spinal cord of EAE mice. The level of TLR8 in monocytes was also reduced after treatment with 1,25-dihydroxyvitamin D3 (Li B. et al., 2013). These results imply that TLR8 plays an accelerating role in EAE and that the inhibition of TLR8 may be a new target for alleviating EAE. However, the specific role of TLR8 in EAE requires further research.

### TLR9

TLR9 is an intracellular TLR that recognizes viral and bacterial nucleic acids in the early phase of infections. The expression of TLR9 in the CNS increases dramatically during the peak of EAE. The activation of APC through TLR9 can overcome tolerance and precipitate EAE (Ichikawa et al., 2002). TLR9 is abundantly expressed on pDCs. CpG DNA, a TLR9 ligand, can strongly induce IFN-α production in pDCs. The treatment of CD4^+^ T cells with CpG DNA can directly enhance their survival (Adamczyk-Sowa et al., 2017). Increased IFN-α can promote the development of the disease. Administration of CpG was found to activate APCs *in vivo*, break immune tolerance of T cells, and induce the onset of EAE in EAE-resistant transgenic B10.S mice (Waldner et al., 2004). Some studies showed that TLR deficiency alleviated EAE. *TLR9*^−/−^ mice developed EAE with a significant delay in disease onset and weak clinical manifestations when compared with WT mice. Absence of TLR9 reduced the degree of inflammation, demyelination, and axonal damage in EAE mice significantly (Prinz et al., 2006). However, other studies have stated that TLR9 may play a tolerogenic role in the development of EAE. Miles et al. (2012) found that B cells treated with chloroquine, a TLR9 inhibitor, *in vitro* had lower IL-10 secretion compared with untreated B cells. Subsequently, in an *in vivo* study, they showed that the spleen cells from EAE in *TLR9*^−/−^ mice secreted significantly less IL-10 than in WT mice.

TLR9 agonists have been used in EAE and MS, but the results are conflicting. Balashov et al. (2010) separated pDCs from RRMS patients and treated these pDCs with TLR9 agonists. They found that treatment with TLR9 agonists increased the secretion of IFN-α (a Th1-promoting cytokine) and that pDCs from IFN-β-treated clinically isolated syndrome (CIS)/RR MS patients had a significantly lower C-terminal TLR9 expression as well as IFN-α, IL-6, and TNF-α secretion compared to untreated patients (Balashov et al., 2010). Ichikawa et al. (2002) induced an EAE-tolerant model by injecting a high dose of PLP139–151 and found that the tolerant function of PLP_139–151_-cultured lymphocyte could be reversed by CpG ODNs. Some studies showed that the administration of bacterial DNA promoted EAE and its clinical symptoms in mice and indicated that TLR9 may play a suppressive role in MS and EAE. Other studies, however, showed opposite results. White et al. found that MIS416 (an agonist target of TLR9) alleviated EAE when administered at the beginning of immunization by suppressing the development of antigen-specific Th1 and Th17 (White et al., 2014). Tao et al. (2016) treated B cells from RRMS patients with a type C CPG/IFN-β combination and found that the treatment enhanced the immunoregulatory cytokine secretion in B cells and suppressed the secretion of inflammatory cytokines in CD4^+^ cells. In addition, the effect of the CPG/IFN-β combination on B cells was significantly higher than that of IFN-β-1a alone (Tao et al., 2016).

### MyD88

MyD88 is a downstream adaptor of the TLR and IL-1 receptor families. MyD88 links TLR family members to IL-1R-associated kinase family kinases. The activation of MyD88 leads to the activation of NF-κB, MAPKs, and activator protein 1 (AP-1; Deguine and Barton, 2014). The activation of these signaling pathways results in the production of pro-inflammatory cytokines such as IL-1, IL-6, and INF-α by activating downstream molecules (Lee et al., 2017). In neurons, IL-1β activation depends on two signaling pathways. The first is MyD88-dependent and results in inflammation. The other is MyD88-independent and has a neuroprotective effect. MyD88 is necessary for the expression of IL-6 and IL-23p40 by splenic mDC and the differentiation of splenic Th17 cells (Marta et al., 2009). MyD88 activation also stimulates the expression of CD40, CD80, CD86, and MHC class II in immature APCs (Deguine and Barton, 2014).

MyD88^−/−^ mice are significantly insufficient in the innate immune system and have no response to ligands specific for TLRs, such as LPS, peptidoglycans, lipoproteins, antiviral compounds, CpG DNA, and flagellins (Prinz et al., 2006). *MyD88*^−/−^ macrophages also fail to produce inflammatory cytokines in response to LPS. Therefore, MyD88-deficient mice have been used extensively as a bacterial pathogen-susceptible model (Kawai and Akira, 2007). As for the EAE model, Prinz et al. found that *MyD88*^−/−^ mice were completely EAE resistant (Prinz et al., 2006). However, MyD88 is dispensable in the pathogenesis of EAE in some specific conditions, since MyD88-deficient PLP TCR transgenic mice developed EAE spontaneously (Wexler et al., 2013).

## Therapy of MS *via* Targeting the TLR-MyD88 Pathway

Since abundant studies have highlighted the specific involvement of TLRs in MS, specific TLR-MyD88-targeting immunotherapy may be applied to the treatment of MS and other inflammatory diseases in the future. Several kinds of TLR antagonists, such as T2.5 and OPN-305 for TLR2, as well as IMO-3100 for TLR7 and TLR9 (Arslan et al., 2012; Wang et al., 2015; Lai et al., 2017), have been produced, and their functions are being improved continuously (Gambuzza et al., 2011).

These TLR-MyD88 signaling pathway inhibitors play their roles in different ways. TLR antagonists have a similar structure with TLR agonists. Some of them are anti-TLR monoclonal antibodies (mAb) or small-molecule antagonists selected from compound libraries. They bind to specific TLR domains, compete with TLR agonists, and inhibit the binding combination of TLR agonists with their receptors as well as blocking signal transduction (Gambuzza et al., 2011). Furthermore, they promote axon sparing and the infiltration of OPCs to repair damaged myelin (Church et al., 2017). The function of TLRs can also be inhibited by soluble TLRs (sTLRs). sTLRs inhibit the functions of TLRs by binding with TLR activators such as LPS and human immunodeficiency virus (Gooshe et al., 2014a).

Since MyD88 is essential for most TLRs (except TLR3 and part of TLR4), inhibition of MyD88 is expected to have a therapeutic effect on MS. For example, the short form of MyD88 (sMyD88) can inhibit TLR-MyD88 signaling by preventing IRAK1 phosphorylation. BB-loop decoy peptides inhibit the TLR-MyD88 signaling pathway by interfering with the MyD88 TIR domain or the full-length MyD88 (Gooshe et al., 2014b).

Several antagonists targeting TLR-MyD88 signaling have been shown to have a therapeutic effect on preclinical models of systemic lupus erythematosus (SLE), rheumatoid arthritis (RA), psoriasis, and colitis (Christensen et al., 2006; Schmidt, 2006). T2.5, a neutralizing antibody against TLR2, has been shown to prevent sepsis induced by TLR2 ligands (Meng et al., 2004). OPN-305, another TLR2-specific monoclonal antibody, inhibited TLR2-mediated pro-inflammatory cytokine production (Arslan et al., 2008). IMO-3100, an inhibitor for TLR7 and TLR9, can reduce the manifestations of the diseases in mouse models such as SLE, RA, psoriasis, and hyperlipidemia (Gambuzza et al., 2011; Suárez-Fariñas et al., 2013). To summarize, even though most of the inhibitors are still in preclinical studies, their application prospects in inflammatory diseases are very extensive. In MS, however, clinical evidence regarding the therapeutic effects of TLR and MyD88 inhibitors is limited. Thus, there is a long way to go before they can be applied in MS treatment.

## TLRs and Other Neuroimmune Diseases

Autoimmune myasthenia gravis (MG) is a neuromuscular disorder characterized by a defective transmission of nerve impulses to muscles. MG is a kind of neuroimmune disease, and about 85% to 90% of MG patients have autoantibodies against the acetylcholine receptor (AChR). Many studies have revealed that MG has a very close relationship with the TLRs-MyD88 signaling pathway. The expression of TLR9 in the PBMCs of patients with MG has a positive relation with clinical severity (Wang et al., 2013). Expression of TLR4 also increased in AChR-specific B cells of patients with MG (Lu et al., 2013). Furthermore, an overexpression of TLR4 was detected in the thymus of MG patients (Bernasconi et al., 2005).

Guillain-Barre syndrome (GBS) is a neuroimmune disease characterized by inflammation and demyelination in the peripheral nervous system (PNS). It is reported that the expressions of TLR2, TLR4, TLR9, MyD88, and NF-κB were elevated in PBMC of patients with GBS (Wang et al., 2012; Du et al., 2015). Some germ proteins, such as LOS sialylation of *C. jejuni* isolates, have clear associations with the development of GBS. The germs activated human DCs *via* TLR4 and induced the production of inflammatory cytokines (Kuijf et al., 2010). In experimental autoimmune neuritis (EAN), an animal model of GBS, the expression of TLR4 and TLR9 in spleen, sciatic nerve, and PBMC was higher than in the control group (Deng and Zhou, 2007). Furthermore, PTX, a drug necessary for EAN establishment in mice, has been demonstrated to work through the TLR4 pathway (Racke et al., 2005). Thus, some neuroimmune diseases, such as MG and GBS, have an association with TLR signaling.

## Conclusion

Although the pathogenesis of MS remains unclear, it is widely considered that the inflammatory response plays a major role in the development of the disease (Adamczyk-Sowa et al., 2017). TLRs are able to recognize specific molecular structures and induce severe inflammatory response by activating downstream signals, such as MAPK and NF-κB (Kawai and Akira, 2007). Several kinds of TLR and MyD88 inhibitors have been developed, some of which have been applied in the therapy of MS, SLE, RA, and colitis. However, the results are controversial, and applications are still in a preclinical phase (Gambuzza et al., 2011). Further investigation is required to confirm the therapeutic effect and the mechanism of TLR-MyD88 inhibition before they can be applied clinically.

## Author Contributions

This manuscript was written by CZ. JC, FC, JZ and TJ modified and edited the manuscript. All authors read and approved the final manuscript.

## Conflict of Interest

The authors declare that the research was conducted in the absence of any commercial or financial relationships that could be construed as a potential conflict of interest.
